# Epidural anesthesia combined with sedation with dexmedetomidine for appendectomy in a patient with amyotrophic lateral sclerosis: a case report

**DOI:** 10.1186/s40981-018-0220-z

**Published:** 2018-12-26

**Authors:** Mikako Kusakai, Atsushi Sawada, Natsumi Kii, Yasuyuki Tokinaga, Naoyuki Hirata, Michiaki Yamakage

**Affiliations:** Department of Anesthesiology, Sapporo Medical University School of Medicine, South 1, West 16, Chuo-ku, Sapporo, Japan

**Keywords:** Amyotrophic lateral sclerosis, Epidural anesthesia, Dexmedetomidine

## Abstract

**Background:**

Patients with amyotrophic lateral sclerosis (ALS) present increased risks for anesthesia-related complications. We present a case of epidural anesthesia combined with sedation with dexmedetomidine for open appendectomy in a patient with ALS who refused invasive mechanical ventilation.

**Case presentation:**

A 50-year-old man with a 3-year history of ALS was scheduled to undergo open appendectomy due to repeated appendicitis. He refused to undergo invasive mechanical ventilation using an endotracheal tube. Hence, we decided to administer epidural anesthesia combined with sedation with dexmedetomidine for anesthesia during the surgical procedure. The patient underwent open appendectomy without complications and with no pain or discomfort during surgery. There were no neurological complications at the 3-month follow-up after surgery.

**Conclusions:**

Epidural anesthesia combined with sedation with dexmedetomidine may be effective for the anesthetic management of patients who would benefit from regional anesthesia.

## Background

Amyotrophic lateral sclerosis (ALS) is a progressive neurodegenerative disease that affects both upper and lower motor neurons and finally leads to death due to respiratory insufficiency [1]. Patients with ALS present increased risks for anesthesia-related complications and require careful perioperative management. General anesthesia may cause fatal respiratory depression because ALS patients are known to be sensitive to neuromuscular blocking agents. Although regional anesthesia is considered to be relatively contraindicated in patients with central nervous system (CNS) disorders, regional anesthesia reportedly did not lead to postoperative worsening of neurologic deficits as compared to preoperative findings in 136 patients with CNS disorders [2]. Here, we report a case of epidural anesthesia combined with sedation with dexmedetomidine for open appendectomy in an ALS patient who refused to undergo invasive mechanical ventilation.

This case is significant for the indication for regional anesthesia and the perioperative anesthetic management, which enabled avoidance of respiratory insufficiency and was not associated with worsening of neurological symptoms at a 3-month postoperative follow-up in our patient with ALS.

## Case presentation

Approval and consent for publication of this report were obtained from the patient and his wife.

A 50-year-old man (body weight 55 kg; height 168 cm) with a 3-year history of ALS was scheduled to undergo open appendectomy due to repeated appendicitis. Clinically, he had advanced bulbar symptoms with dysarthria and dysphagia and muscle weakness in both upper limbs. He had no muscle weakness in his lower limbs. His kidney function was normal. Pulmonary function testing indicated that his forced vital capacity (FVC) was 3.15 L and %FVC was 85.6%. Although he accepted future placement of a percutaneous endoscopic gastrostomy for nutritional supplementation and the necessary equipment for non-invasive mechanical ventilation for pulmonary support, he refused to undergo future invasive mechanical ventilation using an endotracheal tube or tracheotomy in the advanced stages of ALS. In order to avoid the respiratory depression associated with general anesthesia, we decided to administer epidural anesthesia combined with sedation with dexmedetomidine for anesthesia during the surgical procedure.

After obtaining the patient’s informed consent, we transferred him to the operating room and established standard monitoring. After placing the patient in the right lateral position, an 18-gauge Tuohy needle and loss-of-resistance technique with saline were used to obtain epidural access at the T12/L1 vertebral interspace. After careful aspiration to avoid intravascular and intrathecal injection, 11 mL of 1.5% lidocaine with 1:200,000 epinephrine was injected directly via the Tuohy needle. Then, an epidural catheter was placed for intraoperative and postoperative analgesia. We confirmed anesthesia of the T6 to L3 dermatomes using the cold test with ice. Active hip, knee, and ankle flexion were maintained during the surgery. Dexmedetomidine infusion was administered for sedation at 4 μg/kg/h for 10 min and continued at 0.4 μg/kg/h along with 3 L/min O_2_ via a face mask. The patient underwent open appendectomy without complications. The operative time was 43 min. He had no pain or discomfort intraoperatively. Dexmedetomidine infusion was terminated at the end of surgery. For postoperative analgesia, 0.125% levobupivacaine was continuously infused at 4 mL/h via the epidural catheter. He was monitored overnight in the intensive care unit (ICU) to assess his respiratory status and was shifted from the ICU the day after surgery. The patient walked without support after discharge from the ICU. There was no worsening of motor function in his lower limbs or interference with passing urine both during the effect of epidural analgesia and after it had worn off. The epidural catheter was removed on the fourth day after surgery, and the patient was discharged home on the fifth day after surgery. There were no neurological complications due to epidural anesthesia at the 3-month follow-up after surgery.

## Conclusions

ALS is a fatal neurodegenerative disease characterized by death of upper and lower motor neurons. Respiratory muscle weakness occurs due to loss of bulbar, cervical, and thoracic motor neurons [3]. Due to the inherent muscle weakness, general anesthesia combined with muscle relaxants may cause fatal respiratory insufficiency in patients with ALS. Historically, regional anesthesia has been relatively contraindicated in patients with CNS disorders for fear of causing postoperative worsening of neurological symptoms [4]. On the other hand, a previous prospective investigation reported no new or worsening of postoperative neurologic deficits as compared to preoperative findings in 139 patients with preexisting CNS disorders who underwent regional anesthesia [2]. The reduction of FVC has been previously reported to be negligible under lumbar epidural anesthesia in patients without respiratory depression [5]. Although lumbar and low thoracic epidural anesthesia was previously reported to induce a temporary reduction in FVC in a patient with ALS, it was not associated with the development of hypoxia or any clinical symptoms [6]. In our case, the patient had no respiratory depression after epidural anesthesia. In the event that epidural anesthesia severely depressed respiratory function in our patient, we intended to noninvasively support respiration via a ventilation mask until the respiratory depression induced by epidural anesthesia subsided. Therefore, we administered lidocaine as the local anesthetic for epidural anesthesia despite its greater potential to cause motor blockade as compared to other local anesthetic agents, because lidocaine has a shorter duration of action than levobupivacaine and ropivacaine.

Dexmedetomidine is a highly selective α_2_-adrenoceptor agonist. Its clinical effects include sedation, analgesia, and prevention of a detrimental stress response without respiratory depression [7]. Previous randomized trials revealed that dexmedetomidine provided safe and adequate sedation in patients with the risk of pulmonary aspiration who underwent upper endoscopy [8] or endoscopic interventions [9]. Dexmedetomidine provided appropriate sedation and comfort to our patient with ALS without respiratory depression or pulmonary aspiration.

In summary, epidural anesthesia combined with sedation with dexmedetomidine may be effective and suitable for the anesthetic management of certain patients with ALS.

## Abbreviations

ALS: Amyotrophic lateral sclerosis
CNS: Central nervous system
FVC: Forced vital capacity
ICU: Intensive care unit
